# COVID-19 Vaccine Hesitancy Among Older Adults: Evidence From the Medicare Current Beneficiary Survey

**DOI:** 10.1093/geroni/igab046.565

**Published:** 2021-12-17

**Authors:** Divya Bhagianadh, Kanika Arora

**Affiliations:** 1 University of Iowa, IOWA CITY, Iowa, United States; 2 University of Iowa, Iowa city, Iowa, United States

## Abstract

**Objective:**

Older adults have been the most enthusiastic cohort about the COVID-19 vaccine since its rollout. However, there is limited evidence on vaccine hesitancy, particularly among community-dwelling older adults. In this study, we examine the prevalence and predictors (especially information sources) of vaccine hesitancy in this group.

**Methods:**

We use the Medicare Current Beneficiary Survey (MCBS)- Fall 2020 supplement data and employ multivariable logistic regression models to explore this association. We study heterogeneous effects by gender, metro/non-metro residence status, race, and age.

**Results:**

Depending on healthcare providers (HCP), social media, the internet, and family/friends as the main COVID-19 information source was associated with higher odds of negative vaccine intent when compared to those who rely on regular news. We did not find any association of ‘unsure’ vaccine intent and different information sources.

**Discussion:**

Recommendation from an HCP is a strong predictor of any vaccine acceptance and higher negative intent for COVID-19 vaccine among those who depend on HCP for information is concerning. This could be due to vaccine hesitancy among HCPs themselves or due to other mechanisms like infrequent interactions with the health system.

